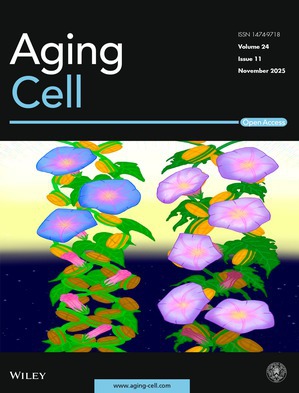# Additional Cover

**DOI:** 10.1111/acel.70290

**Published:** 2025-11-12

**Authors:** Merve Bilgic, Rinka Obata, Vlada‐Iuliana Panfil, Ziying Zhu, Mai Saeki, Yukiko Gotoh, Yusuke Kishi

## Abstract

The cover image is based on the article *Age‐Associated Transcriptomic and Epigenetic Alterations in Mouse Hippocampus* by Merve Bilgic et al., https://doi.org/10.1111/acel.70233.